# Gremlin, A Potential Urinary Biomarker of Anca-Associated Crescentic Glomerulonephritis

**DOI:** 10.1038/s41598-019-43358-5

**Published:** 2019-05-03

**Authors:** Alejandra Droguett, Graciela Valderrama, María E. Burgos, Daniel Carpio, Constanza Saka, Jesús Egido, Marta Ruiz-Ortega, Sergio Mezzano

**Affiliations:** 1 Nephrology Division, School of Medicine, Universidad Austral de Chile, Valdivia, Chile; 2 Cellular Biology in Renal Disease Laboratory, Universidad Autónoma. IIS-Fundación Jiménez Díaz, Madrid, Spain

**Keywords:** Nephrology, Kidney diseases

## Abstract

Gremlin renal overexpression has been reported in diabetic nephropathy, pauci-immune crescentic glomerulonephritis and chronic allograft nephropathy and has been implicated in the pathophysiology of the progression of renal damage. However, it is unknown whether urinary Gremlin can be associated with renal functional status, renal biopsy findings and outcome. To examine these associations we studied 20 patients with ANCA+ renal vasculitis and very high urinary Gremlin (354 ± 76 ug/gCr), 86 patients with other glomerular diseases and moderately elevated urinary Gremlin (83 ± 14 ug/gCr) and 11 healthy controls (urinary Gremlin 11.3 ± 2.4 ug/gCr). Urinary Gremlin was significantly correlated with renal expression of Gremlin (r = 0.64, p = 0.013) observed in cellular glomerular crescents, tubular epithelial cells and interstitial inflammatory cells. Moreover, urinary Gremlin levels were correlated with the number of glomerular crescents (r = 0.53; p < 0.001), renal CD68 positive cells (r = 0.71; p < 0.005), tubulointerstitial fibrosis (r = 0.50; p < 0.05), and serum creatinine levels (r = 0.60; p < 0.001). Interestingly, Gremlin expression was colocalized with CD68, CD163 (monocyte/macrophage markers) and CCL18 positive cells. ROC curve analysis showed that the cutoff value of urinary Gremlin in glomerular diseases as 43 ug/gCr with 72% of sensitivity and 100% of specificity [AUC: 0.96 (CI 95% 0.92–0.99] (p < 0.001). For ANCA+ renal vasculitis the value of urinary Gremlin of 241 ug/gCr had 55% of sensitivity and 100% of specificity [AUC: 0.81 (CI 95% 0.68–0.94) (p < 0.001]. Based on these results we propose that urinary Gremlin represents a non-invasive biomarker in ANCA+ renal vasculitis, and suggest a role of Gremlin in the formation of crescents.

## Introduction

Gremlin was identified as one of developmental genes, re-expressed during renal damage that could play a role in the progression of diabetic nephropathy and other chronic renal diseases^1^.

Gremlin belongs to a family of bone morphogenetic proteins (BMPs) antagonists^2–4^ that have a well-recognized role in organogenesis and fibrotic related disorders^5–7^ Gremlin is induced by TGF-β in renal cells^2^, and it is a downstream mediator of TGF-β, activating the Smad pathway and inducing epithelial-mesenchymal transition^8^. Moreover, in a BMP-independent manner, Gremlin could contribute to renal inflammation via vascular endothelial growth factor receptor 2 pathway^9^.

Gremlin knockout mice die of renal aplasia, lung defects and limb malformations^7,10–14^, and we have recently shown that Gremlin tubular overexpression aggravates folic acid-induced and streptozotocin-induced renal damage^15,16^.

In human diabetic nephropathy, Gremlin is most prominently expressed in podocytes and areas of tubulointerstitial fibrosis, colocalized with TGF-β expression and correlated with tubulointerstitial score damage^17,18^. Gremlin has also been observed in various nephropathies, notably in pauci-immune crescentic vasculitis where strong expression of Gremlin mRNA and protein were observed at glomerular crescents, and in tubular and infiltrating interstitial cells^19^. However, it is not known if Gremlin is present in the urine during kidney injury and whether it could be a novel biomarker with diagnostic or prognostic value in glomerular diseases. The present study was done to answer these questions.

## Material and Methods

### Patients studied

We studied 106 consecutive patients with a glomerular disease, admitted for a diagnostic renal biopsy. 20 of them with ANCA-associated pauci-immune glomerulonephritis (ANCA CGN), 26 Systemic Lupus Erythematosus (SLE), 18 Membranous Nephropathy (MGN), 23 patients with IgA nephropathy (IgAN), and 19 Focal Segmental Glomerulosclerosis (FSGS).

**Figure 1:**
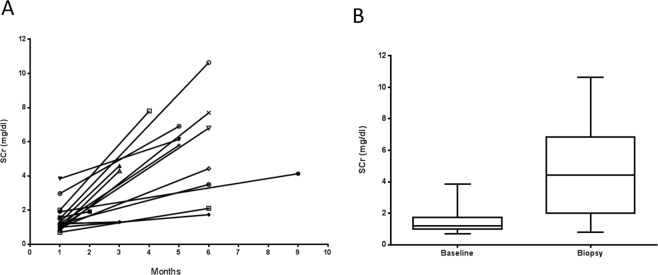
Increase of serum creatinine (mg/dl) before renal biopsy in patients with ANCA+ Crescentic Glomerulonephritis. (**A**) 17 patients had a baseline mean serum creatinine (SCr) (mg/dl), of 1.50 ± 0.8, evaluated at different time points (one to 9 months) prior to the kidney biopsy. (**B**) At the time of the biopsy, the mean serum creatinine was 4,7 ± 2.6, with a median of 4.4 (IQR 4.8) (Wilcoxon p = 0.001).

Clinical and demographic data of these patients are presented in Tables 1 and 2, and Fig. 1.

**Table 1:** Clinical and demographic data of patients with ANCA + CGN.

ANCA CGN Patient	Age (years)	Gender (F/M)	Urinary Gremlin (ug/gCr)	Serum Creatinine (mg/dl)	Crescent (%)	Masson (% staining area)	ANCA		URINALYSIS		
							IFI	ELISA (U/ml)	Prot (mg/dl)	RBC (cells/field)	Casts
1	41	F	1408	4.1	46	4	c	5 PR3	75	0–2	(—)
2	81	F	36	1.9	29	2.7	p	>100 MPO	75	10–20	(—)
3	83	F	665	4.6	67	1.8	p	>100 MPO	150	20–30	hyaline granular
4	63	F	333	6.1	86	1.6	p	20 MPO	500	5–10	hyaline
5	68	F	78	1.7	38	2.0	p	9 MPO	500	5–10	hyaline
6	72	F	413	10.6	58	10.9	p	93 MPO	150	80–90	granular
7	67	M	892	7.8	75	2.1	p	>100 MPO	300	20–50	hyaline
8	33	F	644	5.8	23	8.7	p	73 MPO	150	20–50	RBCs granular
9	60	M	417	4.3	30	8.4	p	9 MPO	300	20–30	(—)
10	56	F	362	6.8	40	5.1	p	>100 MPO	300	10–20	RBCs
11	64	F	292	4.3	63	5.5	p	10 MPO	500	0–2	hyaline
12	59	M	490	3.5	50	10.8	p	nt	150	60–70	(—)
13	60	F	75	1.3	36	7.1	p	84 MPO	150	10–20	hyaline granular
14	67	M	132	5.8	100	0.3	p	76 MPO	75	80–90	(—)
15	56	M	59	0.8	40	2.8	p	18 MPO	75	80–90	hyaline granular
16	21	F	159	0.9	40	15.8	p	nt	75	20–50	hyaline
17	69	F	96	7.7	50	2.8	p	9 MPO	150	20–50	granular
18	60	M	168	6.9	50	6.8	p	9 MPO	100	30–40	(—)
19	29	F	256	2.1	100	4.0	p	38 MPO	75	20–50	hyaline granular
20	61	M	100	7.9	71	5.8	c	7.4 PR3	30	20–30	(—)

nt:non tested, RBC:red blood cells, MOP:Mieloperoxidase, PR3:proteinase 3.

**Table 2:** Clinical and demographic data of patients studied.

	n	Gender (M/F)	Age x (years)	Serum Creatinine (mg/dl)	Proteinuria (mg/dl)
ANCA CGN	20	7/13	58.5 ± 3.6	4,6 ± 0.6	194.0 ± 34.3
SLE	26	5/21	34.3 ± 2.2	2.0 ± 0.4	342.8 ± 68.5
MGN	19	7/12	49.6 ± 3.3	0.83 ± 0.1	442.6 ± 48.5
IgAN	23	12/11	36.4 ± 3.1	1.2 ± 0.1	102.2 ± 52.5
FGS	18	8/10	45.1 ± 3.9	1.4 ± 0.2	298.9 ± 44.2
NORMAL	16	4/12	36.8 ± 3.2	nd	0

All the patients with ANCA+ crescentic glomerular disease were developing a rapidly progressive renal failure (Fig. 1), with history of microhematuria, dysmorphic red blood cells and/or red cell casts and with 23 to 100% of glomerular crescents. We defined as a non-ANCA crescentic glomerulonephritis (non-ANCA CGN), a subgroup of 20 patients with SLE or IgA nephropathy, with >25% of glomerular crescents.

### Human renal biopsies

Kidney samples were obtained by percutaneous renal biopsy performed at the Division of Nephrology, Austral University, Valdivia, Chile. The samples were studied after informing and obtaining the patient written consent and the approval by local hospital ethics committee (Comité de Ética de Investigación, Servicio de Salud Valdivia, Ministerio de Salud, Chile ORD-No. 120/2016). The study was in adherence with the Declaration of Helsinki. Light microscopy, immunofluorescence (IF) and electron microscopy studies were performed using routine methods for diagnostic use.

### Urine samples

Urine samples from patients were collected the same day and prior to renal biopsy. Samples were immediately centrifuged for 15 minutes at 3000 rpm and the supernatants stored at −80 °C. Urinary Gremlin was determined no later than 3 month after collection avoiding repeated freeze-thaw cycles. Gremlin was determined using an enzyme-linked immunosorbent assay with biotin-conjugated antibody specific to GREM1, followed by avidin conjugated to horseradish peroxidase. The sensitivity or lower limit of detection is less than 50 pg/ml and the intraassay and inter-assay coefficient of variability (CV) are <10% and <12%, respectively (Uscn Life Science Inc., Wuhan, China). Urinary and serum creatinine were measured with Creatinine Liquicolor Kit (Human Gesellschaftfür Biochemica und DiagnostikambH, Wiesbaden, Germany) and Cobas CREJ2 (Roche Diagnostics Limited, Mannheim, Germany), respectively.

### Immunohistochemistry (IHC)

Paraffin-embedded renal sections were used for detection of Gremlin, CD163, CD68 and CCL18. The following primary antibodies were employed: rabbit polyclonal anti-Gremlin (dilution 1:300, ABGENT, AP6133a, San Diego CA, USA); rabbit polyclonal anti-CCL18 (dilution 1:100, Sigma-Aldrich, Saint Louis, MO USA); mouse monoclonal anti-CD163, monocyte/macrophage marker (dilution 1:500, CELL MARQUE, Rocklin CA, USA) and mouse monoclonal anti-CD68 (Kp-1) monocyte/macrophage marker (dilution 1:500, CELL MARQUE). IHC was performed following heat-induced epitope retrieval (HIER) [microwaving for 10 min in citrate buffer for Gremlin and CCL18 and Trilogy (CELL MARQUE) for CD163 and CD68]. After blockage endogenous peroxidase activity and non-specific-background with Power Block (Biogenex, Fremont CA, USA), the slides were incubated with primary antibody overnight at 4 °C and detected with Immpress Reagent (Vector, Burlingame CA, USA). The reaction was developed with DAB (SK 4105, Vector) and counterstained with hematoxylin.

Image analysis and quantification of the IHC signals were performed using the KS300 imaging system, version 3.0 (Zeiss). For each sample, the mean staining area was obtained by an analysis of 20 fields (x20). The staining score is expressed as square millimeters per density.

### *In situ* hybridization (ISH)

It was performed as previously describedfor Gremlin^18^, using the following antisense Gremlin probes: 5′-TGAAAGGAACCTTCCTCCTTCC3′, 5′-ATGGGAGAGCACTGGATCAAAA-3′ and 5′-CAGGCACTGACTCAGGAAGACA-3. The specificity of the reaction was confirmed by RNAse treatment, using a sense probe, or without probe.

### Statistical analysis

Statistical analysis was conducted using SPSS Statistics version 20 and Graphpad Prism 7. Numerical variables are listed as mean and standard deviation or median and interquartile range. Mann-Whitney and Kruskal-Wallis tests were used to compare urinary Gremlin between different renal pathologies and healthy controls. Wilcoxon test to compare evolutive changes of serum creatinine and urinary Gremlin. Spearman test was used to correlate urinary Gremlin and crescent percentage, serum creatinine, tisular Gremlin and TIF. Receiver operating characteristic (ROC) curves and Youden´s index were performed to determine the cut-off point, sensitivity and specificity of urinary Gremlin in renal pathology and pauci-immune crescentic GN. Area under the curve (AUC) was used to assess the diagnostic value and was reported with 95% CIs. p values < 0.05 were considered statistically significant.

### Compliance with ethical standards

The samples were studied after informing and obtaining the patient written consent and the approval by local hospital ethics committee (Comité de Ética de Investigación, Servicio de Salud Valdivia, Ministerio de Salud, Chile). The study was in adherence with the Declaration of Helsinki.

## Results

### Urinary Gremlin levels are elevated in pauci-immune crescentic glomerulonephritis

**Figure 2:**
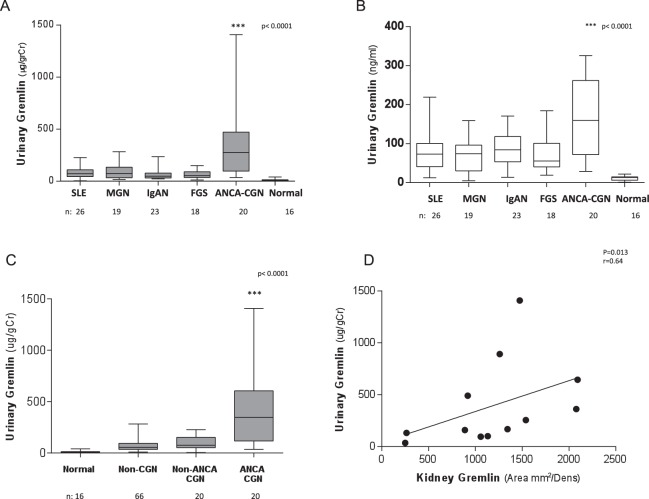
Evaluation of urinary Gremlin in glomerular diseases. (median and interquartile range). (**A**) Urinary Gremlin adjusted by urinary creatinine (ugGr/grCr) was significantly higher in patients with ANCA − CGN versus patients with other glomerular diseases (p < 0.0001). (Mdn = 274, IQR = 375 ug/grCr). (**B**) Unadjusted Urinary Gremlin observed confirming a significant higher median values for ANCA vasculitic patients. (Mdn = 159,5, IQR = 190) (**C**) Gremlin levels were still markedly and significantly higher in ANCA + CGN (n = 20) (Mdn = 274, IQR 375) compared with non-vasculitic GN with crescents (n = 17 SLE, 3 IgAN) (Mdn = 75, IQR 106) and other glomerular diseases without crescents (non CGN n = 66) (Mdn = 56, IQR 59 ug) and healthy donors (Mdn 9.35, IQR 7.7 ug/gCr) (p < 0.0001). (**D**) Spearman correlation between Gremlin protein staining observed by immunohistochemistry (IHC) in renal biopsies and urinary Gremlin excretion expressed as ug/grCr (n = 12) (r = 0.64, p = 0.013).

Urinary Gremlin levels adjusted and not adjusted by urine creatinine, were significantly higher in patients with ANCA-crescentic glomerulonephritis than in patients with other glomerular diseases (p < 0.0001) (Fig. 2A,B).

In order to define if these increased Gremlin values were only related to the presence of glomerular crescents, we compared the values in ANCA-crescentic glomerulonephritis with other glomerulopthies (IgA and SLE nephropathy) that presented crescents in more that 25% of the glomeruli. Urinary Gremlin levels were significantly higher in ANCA CGN (n = 20, 354 ± 76 ug/gCr) than those found in non-ANCA CGN in SLE (n = 17) and in IgA nephropathy (n = 3) that were 95.1 ± 15.2 ug/gCr). The urinary levels of Gremlin were very much lower in other non-crescentic renal diseases (Non CGN n = 66, 72.3 ± 6.8 ug/gCr) and healthy donors (11.3 ± 2.4 ug/gCr) (p < 0.0001) (Fig. 2C).

**Figure 3:**
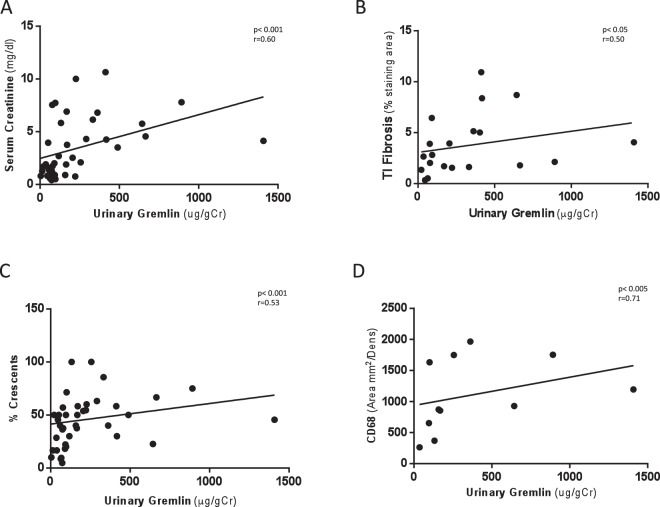
Spearman correlation of urinary Gremlin levels with indices of renal damage in patients with CGN. We evaluated the relation between urinary Gremlin and different markers of renal damage. There was a significant correlation between urinary Gremlin and (**A**) Serum creatinine (p < 0.001 r = 0.60). (**B**) Tubulointerstitial fibrosis evaluated by Masson staining (p < 0.05 r = 0.50). (**C**) Percentage of glomerular crescents (%) (r = 0.53, p < 0.001) and (**D**) Presence of macrophages (CD68 positive cells) (Area mm^2^/Dens) (r = 0.71, p < 0.005) observed in kidney biopsies of patients with CGN.

These values were correlated with tisular Gremlin protein measured by immunohistochemistry (IHC) in renal biopsy (p = 0.013 r = 0.64) (Fig. 2D). Next, we evaluated the relation between urinary Gremlin and different markers of renal damage in patients with ANCA vasculitis. and found there is a strong correlation between urinary Gremlin (ug/gCr) and serum creatinine (mg/dL) (p < 0.001, r = 0.60) (Fig. 3A), tubulointerstitial fibrosis evaluated by Masson staining (% staining area) (p < 0.05, r = 0.50) (Fig. 3B), number of crescents (%) (p < 0.001, r = 0.53) (Fig. 3C) and presence of macrophages CD68 positive cells (area mm^2^/Dens) (p < 0.005, r = 0.71) (Fig. 3D).

Finally, in order to reject the hypothesis that the difference in urinary Gremlin was only attributed to the number of crescents, we compared patients from both groups, vasculitic and non vasculitic glomerular diseases (15 each group) with a similar number of crescents, observing a strongest amount of urinary Gremlin only in ANCA positive patients (358 ug/gCr vs 102 ug/gCr) (Table 3).

**Table 3:** Urinary Gremlin was significantly increased in crescentic ANCA vasculitis versus crescentic non vasculitic.

	ANCA-CGN (n = 15)		SLE-CGN (n = 15)	
	Urinary Gremlin (ug/gCr)	% crescents	Urinary Gremlin (ug/gCr)	% crescents
Mean	358	43	102	34
e.s.m	93	3.2	25	4.9
median	292	40	86	30

### Receiver-operating characteristic (ROC) analysis

**Figure 4:**
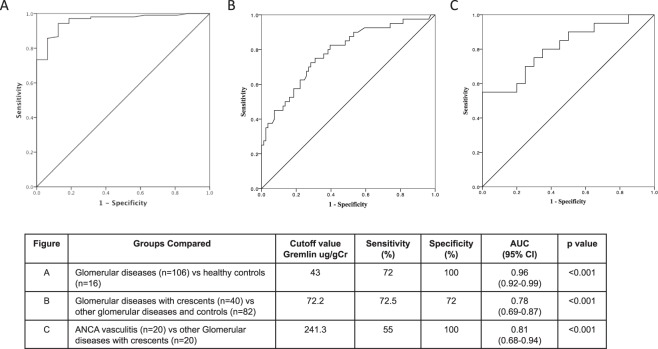
Receiver-operating characteristic (ROC) analysis of urinary Gremlin in patients with different glomerular diseases. In ROC curves we reported (**A**) cutoff value of 43 ug/gCr for patients with glomerular diseases with 72% of sensitivity and 100% of specificity. AUC: 0.96 (95% CI 0.92–0.99) (p < 0.001). (**B**) In patients with glomerular diseases with crescents, we reported a cutoff value of 72.2 ug/gCr of urinary Gremlin with 72.5% of sensitivity and 72% of specificity. AUC 0.78 (95% CI 0.69–0.87) (p < 0.001). (**C**) In patients with ANCA Vasculitis we reported a cutoff value of 241.3 ug/gCr with 55% of sensitivity and 100% specify. AUC: 0.81 (CI 95% 0.68–0.94) (p < 0.001).

In the ROC curves generated to predict accuracy of urinary Gremlin to be a non-invasive biomarker for patients with renal pathology, we reported a cutoff value of 17 ug/gCr of urinary Gremlin for healthy controls and 43 ug/gCr as a cutoff value for patients with glomerular diseases (n = 106) with 72% of sensitivity and 100% of specificity; AUC: 0.96 (CI 95% 0.92–0.99) (Fig. 4A). Also, in a similar manner, in patients with glomerular diseases with crescents (ANCA vasculitis and non-vasculitis) (n = 40) we reported a cutoff value of 72.2 ug/gCr with 72.5% of sensitivity and 72% of specificity, AUC: 0.78 (CI 95% 0.69–087) (Fig. 4B), and finally in patients with ANCA Vasculitis (n = 20) we reported a cutoff value of 241.3 ug/gCr of urinary Gremlin with 55% of sensitivity and 100% of specificity. AUC: 0.81 (CI 95% 0.68–0.94) (p < 0.001) (Fig. 4C). These curves predict the ability of urinary Gremlin to be a non-invasive biomarker for patients with this renal pathology.

#### Follow up study of urinary Gremlin in patients with ANCA + CGN

**Figure 5:**
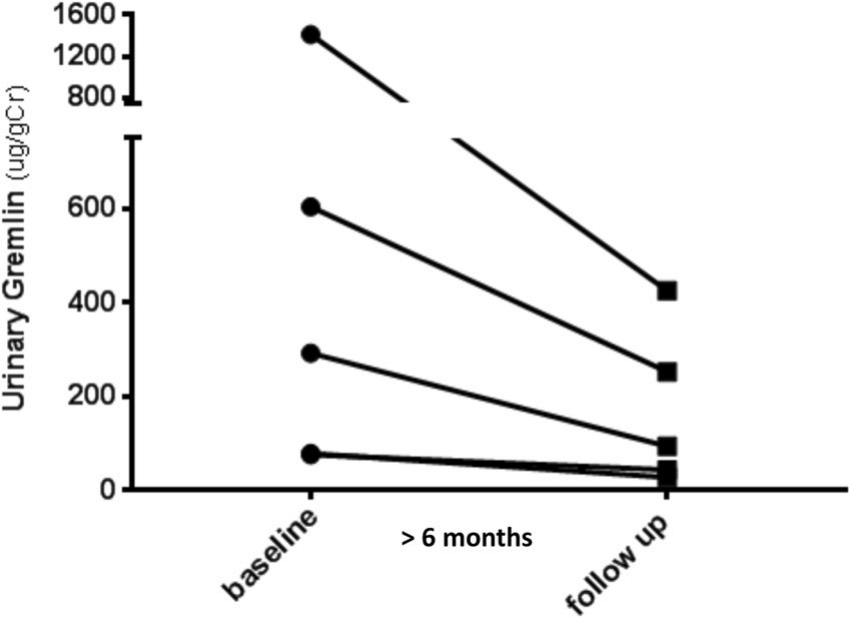
Follow up study of urinary Gremlin in patients with ANCA + CGN. In 5 patients, while in remission, post induction therapy, there was a significant decreased of Urinary Gremlin levels, (ug/gCr), mainly observed in those patients with severe histologic activity. (Mean ± SEM = 499 ± 249 versus 168 ± 75; Mdn 292 versus 93; Wilcoxon p < 0.001).

All the patients received an induction therapy with corticosteroids, cyclophosphamide or rituximab, by three or six months. Follow-up determinations of urinary Gremlin were carried out after induction therapy in a small cohort of patients, while in remission. As observed in Fig. 5, those patients with a particularly active disease at the beginning, showed a significant reduction of urinary Gremlin during remission. (499 ± SE 249 vs. 168 ± 75; median 292 vs. 93 ug/grCr; p < 0.001).

### Renal Gremlin

**Figure 6:**
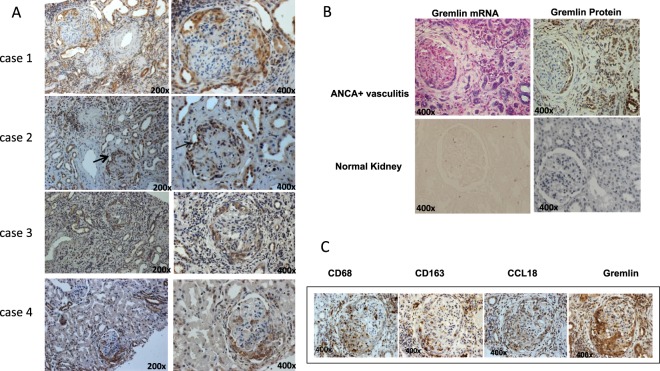
Localization of Gremlin and inflammatory markers in crescentic glomerulonephritis. (**A**) In renal biopsies from patients with ANCA associated CGN (case 1 and 2), Gremlin protein was detected at glomeruli in cellular crescents, at tubular cells, and interstitial infiltrating cells, mainly monocytes-macrophages (arrows). In patients with IgA (case 3) and Lupus Nephritis (case 4), Gremlin protein was mainly detected in cellular crescents and tubular cells. (**B**) By *in situ* hybridization, Gremlin mRNA was mainly detected in glomerular podocytes and parietal epithelial cells, and interstitial tubular cells. In serial sections of a representative case of ANCA + CGN a clear co-localization between Gremlin protein and mRNA was found in all renal structures. Gremlin protein and mRNA is not observed in Normal Kidney. (**C**) There was a clear co-localization between Gremlin protein staining and CD68, CD163 and CCL18 as activate monocyte/macrophages markers respectively. Immunohistochemistry was done in serial sections of renal biopsy from patients with ANCA + CGN. Figure C shows a representative case of at least 10 studied. Magnification 200x and 400x, as indicated.

Renal Gremlin protein expression was evaluated by immunohistochemistry in 35 patients with glomerular diseases with crescents (ANCA+ vasculitis and non vasculitis). In 12 patients with pauci-immune crescentic glomerulonephritis, Gremlin was mainly detected in areas with cellular crescents, tubular cells and interstitial inflammatory cells (arrows) (Fig. 6A patients 1 and 2). Also, Gremlin staining was detected in crescents and tubular cells in IgA nephropathy and SLE Class IV GN, but with lower intensity than ANCA+ vasculitis (Fig. 6A patients 3 and 4). In these patients Gremlin mRNA was confirmed by ISH in crescentic proliferative cells, tubular epithelial cells, and interstitial inflammatory cells. In serial sections of a representative case of ANCA+ vasculitis (12 patients studied) a clear co-localization between Gremlin protein and mRNA was observed (Fig. 6B). As noted, tisular Gremlin was significantly correlated with urinary Gremlin (Fig. 2D).

### Gremlin and Inflammation markers

Monocytes/macrophages play an important role in active glomerular inflammation and may be detected by different phenotypic markers^20^. Tissue macrophages are divided into the M1 and M2 subtypes according to the expression of different surface molecules and transcription factors. As it has been proposed that macrophages can directly transdifferentiate into myofibroblasts through a macrophage-myofibroblast transdifferentiation process^21^, we evaluated macrophage infiltration by identifying CD68+ cells (specific pan-monocyte/macrophage marker) as well as CD163+ cells, a marker that is increased in M2 macrophages^22^.

In addition, macrophages and dendritic cells in the kidney were identified as CCL-18 producing cells, that are known to drive renal inflammation in ANCA-associated crescentic GN^23^. Previously, we have shown that Gremlin expression is also induced in human monocytic cells stimulated *in vitro* by TGF-β^19^.

In the present study, in patients with ANCA + CGN we observed a strong expression of Gremlin protein in cellular crescents and interstitial inflammatory cells that showed co-expression of CD163, CCL-18 and CD68 (Fig. 6C).

## Discussion

In this prospective study we have demonstrated for the first time that Gremlin may represent a new biomarker of ANCA-associated renal vasculitis which can be linked directly to the underlying pathophysiology of this glomerular disease. High urinary levels of Gremlin are associated with a more severe disease activity as represented by the number of glomerular crescents, tubulointerstitial fibrosis and interstitial inflammation. Furthermore, in this study we observed a close correlation between kidney and urinary Gremlin and a significant positive correlation between the serum creatinine and urinary Gremlin, at the beginning and in the follow up study of these patients.

Although our observation was made in a relative modest number of patients with ANCA+ crescentic GN, the ELISA employed and range of urinary Gremlin values in our study are in agreement with those reported by Afkarian *et al*.^24^, in a group of patients with diabetic nephropathy.

ANCA-associated vasculitis is the major cause of rapidly progressive GN, and despite advances in our understanding of the pathogenesis of the disease, renal outcomes remain poor in a considerable percentage of patients^25^. The cellular crescent formation results from disruption of glomerular capillaries that allows inflammatory mediators and infiltrating cells to enter Bowman´s space, where they induce activation and proliferation of parietal epithelial cells (PECs), monocytes/macrophages and fibroblasts infiltration^26^. Previously, we have already reported strong expression of Gremlin mRNA and protein in cellular and fibrocellular crescents, particularly in proliferating parietal epithelial cells and monocytes, suggesting a pathogenic role of Gremlin in crescents formation^19^. It is important to note, that with a comparable percentage of mainly fibrocellular glomerular crescents (43 ± 3 versus 34 ± 5), patients with pauci immune disease had markedly higher urinary levels of Gremlin than patients with lupus nephritis or IgA nephropathy (298 ug/gCr versus 86 ug/gCr).

The role of Gremlin as a BMP antagonist has clearly been demonstrated in fibrotic related disorders, either acting as an inhibitory trap protein for BMP-7 or directly as a downstream mediator of TGF-β^1,7^. Experimental studies have shown that Gremlin contributes to renal fibrosis, as confirmed *in vitro* by its role in the regulation of fibroblasts proliferation and matrix production and in the induction of epithelial to mesenchymal transition of tubular epithelial cells^1^. More recently, we have demonstrated that Gremlin is a ligand of vascular endothelial growth factor receptor 2 in tubular epithelial cells, and participates in the regulation of renal inflammation by activation of the Nuclear Factor-KB pathway and chemoattraction for monocytes/macrophages through induction of MCP-1^9^. These considerations led us to speculate that urinary Gremlin shed by crescent macrophages could correlate with active glomerular inflammation and participate in the interstitial inflammatory response.

Infiltrating macrophages are found in all renal diseases. The role of macrophages in the progression of renal damage is attracting special interest. Although M1 macrophages are more pro inflammatory and M2 macrophages have been suggested to exert anti-inflammatory and tissue repair properties, important questions regarding role of M1/M2 in acute phase and in the transition to fibrosis or recovery are still unresolved. Previous studies have observed that M2 macrophages are involved in the pathogenesis of acute tubulointerstitial lesions in patients with crescentic glomerulonephritis^22^, and other acute renal diseases, as recently observed in Sjogren’s disease, were CD163-positive macrophages are positively correlated with urinary NAG and β2-microglobulin^27^.

We have observed that in patients with ANCA + CGN the expression of Gremlin is localized in CD68, CD163 and CCL-18 positive cells, supporting the important role of macrophages, including M2 macrophages, in the pathogenesis of ANCA-associated crescentic GN. Previous studies have shown that urinary excretion of soluble CD163 (sCD163) is a biomarker of macrophage activation in rats with experimental vasculitis and in patients with small vessel vasculitis and lupus nephritis^20,28^. Urinary sCD163 levels are elevated in active renal vasculitis, compared with patients with active extrarenal vasculitis and all patients in remission^20^. We require further studies to validate Urinary Gremlin, as a comparable biomarker in those clinical conditions.

The CC chemokine ligand 18 (CCL18), acting through CC chemokine receptor 8 (CCR8) on mononuclear cells, was identified as the most upregulated chemotactic cytokine in patients with ANCA-associated vasculitis and correlated with crescent formation, interstitial inflammation, and impairment of renal function. Serum CCL18 levels were higher in patients with renal ANCA disease compared with patients with other forms of crescentic GN^23^. The localization of Gremlin in CCL-18, CD163, and CD68 positive cells suggests a key role of activated macrophages on the crescent formation and in interstitial inflammation.

Limitations of our work include that selection and study of our patients are from a single center and the number of patients and the follow-up were limited. The modifications of urinary Gremlin by therapeutic interventions need to be confirmed in a larger number of patients, specially since only 2 out of 20 cases are pattern C-ANCA (PR3 isotype). Therefore, additional studies are needed to confirm these results that potentially may be important in clinical practice.

In conclusion we have identified a highly specific association between ANCA+ crescentic glomerulonephritis and the level of urinary Gremlin. This protein is strongly expressed in glomerular crescents, as macrophages marker and the shedding of Gremlin directly into the urine makes it very attractive as a potential urinary biomarker of renal vasculitis.
